# Pain Scores Are Not Predictive of Pain Medication Utilization

**DOI:** 10.1155/2011/987468

**Published:** 2011-10-11

**Authors:** Suzanne Galloway, Maryann Chimhanda, Jayme Sloan, Charles Anderson, James Sinacore, Linda Brubaker

**Affiliations:** Department of Obstetrics and Gynecology, Loyola University Medical Center, 2160 S. First Avenue, Maywood, IL 60153, USA

## Abstract

*Objective*. To compare Visual Analogue Scale (VAS) scores with overall postoperative pain medication requirements including cumulative dose and patterns of medication utilization and to determine whether VAS scores predict pain medication utilization. *Methods*. VAS scores and pain medication data were collected from participants in a randomized trial of the utility of phenazopyridine for improved pain control following gynecologic surgery. *Results*. The mean age of the 219 participants was 54 (range19 to 94). We did not detect any association between VAS and pain medication utilization for patient-controlled anesthesia (PCA) or RN administered (intravenous or oral) medications. We also did not detect any association between the number of VAS scores recorded and mean pain scores. *Conclusion*. Postoperative VAS scores do not predict pain medication use in catheterized women inpatients following gynecologic surgery. Increased pain severity, as reflected by higher VAS scores, is not associated with an increase in pain assessment. Our findings suggest that VAS scores are of limited utility for optimal pain control. Alternative or complimentary methods may improve pain management.

## 1. Introduction

Optimal postoperative pain control has been identified as a potentially important modifier of both short- and long-term surgical outcomes [1]. Uncontrolled post operative pain has been linked to loss of respiratory mechanics [2, 3] and cardiac morbidity through activation of the sympathetic nervous system, coagulation cascade, and surgical stress response [4, 5]. Suppression of this stress response via pain control has been suggested to decrease risk of post operative ileus through reduction of the inflammatory response to surgery [6, 7]. Post operative pain may be a major determinant of surgical wound infection, as shown with data by Akca et al. [8] revealing increased subcutaneous oxygen partial pressures and perfusion to tissue in patients with better pain control than patients with inadequate pain control.

Quality of life in the post operative period is dependent on adequate pain relief. Effective pain control in the early post operative period may also lead to clinically important benefits for long-term recovery, including less chronic pain [9, 10]. The association between the intensity of acute post operative pain and later development of chronic pain has been demonstrated after inguinal hernia repair [11], breast surgery [12], and thoracotomy [13].

Patient satisfaction and perception of quality of care has been shown to be linked to provider efforts to optimize pain control. A 2008 survey conducted by the Joint Commission on Accreditation of Healthcare Organizations (JCAHO) reported a clear relationship between responses to patient's overall satisfaction with pain management and their perceptions of pain assessment frequency, response time, and concern demonstrated by staff [14]. This study also demonstrated that patients' actual pain severity score did not relate to perceived overall satisfaction with pain management.

Visual Analogue Scales (VAS) have been utilized in multiple clinical and experimental settings to measure pain [15] and have been demonstrated to be reliable [16–18], generalizable [19, 20], and internally consistent measures of pain sensation and intensity [16, 19] as well as sensitive measures of effects of analgesic treatments [21–24]. Though VAS scores are used in a variety of situations to compare pain treatments, there is no report in the literature comparing mean VAS score and pain medication utilization or mean VAS score and frequency of documented post operative pain assessment. 

As a planned secondary analysis of a registered randomized controlled trial, we compared mean VAS and post operative utilization to determine whether VAS scores predict pain medication utilization. In addition, we explored the relationship of pain severity, as reflected by the VAS score, and the frequency of pain assessment. We hypothesized that patients with higher mean VAS scores would have more frequent VAS assessments and higher pain medication utilization.

## 2. Materials and Methods

Data for this planned secondary analysis was collected as part of the Catheter Analgesia (CATH) study, an IRB approved, randomized trial, registered at clinicaltrial.gov (NCT00771173). The primary study was designed to determine whether phenazopyridine improved pain in catheterized inpatients recovering from gynecologic surgery. Two hundred and nineteen women were assigned 1 : 1 to placebo or phenazopyridine HCl between September 2008 and September 2009. Group assignment was masked by instillation of orange dye in the Foley bag of both groups. 

Eligible candidates were adult women undergoing gynecologic surgery who required an indwelling catheter for a minimum of 12 postoperative hours and who could tolerate oral pain medications within 12 post operative hours. Criteria for exclusion included hypersensitivity to phenozopyridine, hepatic failure or other known contraindications to phenazopyridine, suprapubic catheterization, inability to take oral medication within 12 hours after surgery, and pregnancy. Patients with intraoperative urologic injury were excluded.

Randomized participants received either 200 mg phenazopyridine or placebo oral capsule every eight hours while Foley catheter in place or for 24 hours post operatively, whichever came first. Group assignments were known only to the research pharmacist who randomized subjects immediately following completion of surgery. Randomization was performed utilizing sequentially numbered opaque envelopes generated by a randomized block permutation. Surgical pain was clinically treated without standardization. 

The primary outcome was mean VAS measurements during the postoperative period according to standard clinical order sets. The clinical VAS scores were obtained during clinical care by nurses, residents, and anesthesiologists. The investigators later abstracted the data from the electronic medical record (EMR). Randomized participants who did not have at least one VAS score recorded were not included in the analysis. 

Results from the primary project have been reported separately [25]. Data frequencies and regression were performed with SPSS-pc, Version 16.0 [26]. We combined the two groups for the statistics comparing both VAS and post operative pain medication utilization as well as VAS frequency with mean VAS scores.

## 3. Results and Discussion

The demographic data and surgery type for the 219 participants are presented in Table 1. Most participants were Caucasian (80%); the mean age was 55 years old. Surgical groups included: abdominal procedures (83%), laparoscopic procedures (35%), and vaginal surgery (20%). Patients could be assigned to both a laparoscopic group as well as the abdominal group. 

The mean length of study participation was eighteen hours during which 187 patients (86%) had at least one VAS recorded.  Table 2 presents the VAS frequency data for the total of 647 VAS assessments available for analysis.

The mean VAS score was 2. Most VAS scores (86%) were <2. Approximately 1 in 5 (22%) participants reported that their highest VAS score was zero. We did not detect clinical or statistical differences in the mean VAS scores for the study group compared to the placebo group (*P* = 0.820) [25].

**Figure 1:**
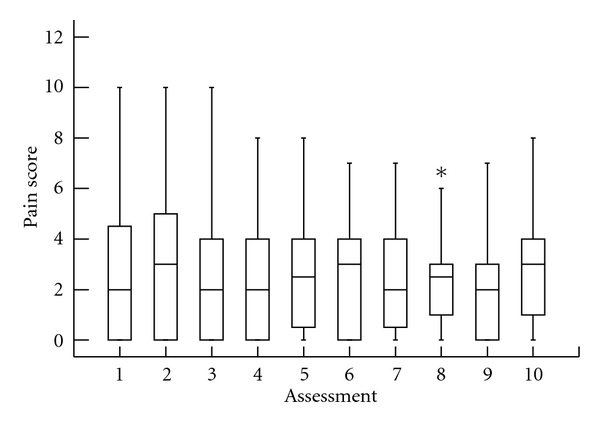
VAS score distribution over time: (note: time intervals were not standardized). Early (VAS1) to later (VAS 10) assessment: VAS 1 represents the earliest chronological assessment after surgery, with VAS 2 and forward representing subsequent assessments later in the postoperative period.

Figure 1 presents the distribution of each VAS score assessment. There was no association when comparing time and VAS score. 

**Figure 2:**
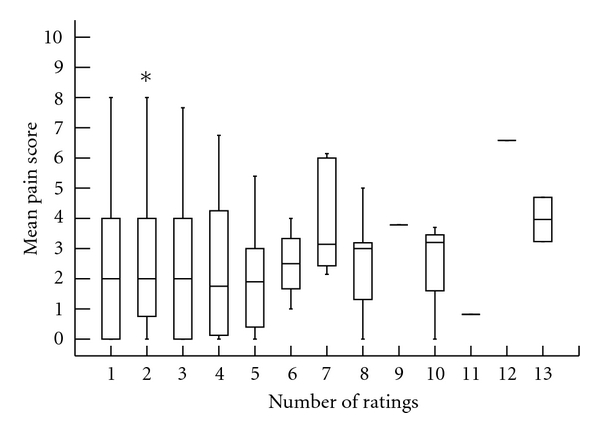
Number of VAS scores recorded and mean VAS.

VAS score frequency, meaning the number of scores recorded, and mean VAS scores are compared in Figure 2. Pain assessment frequency was not predictive of mean VAS score (*r* ^2^  .006). 

**Figure 3:**
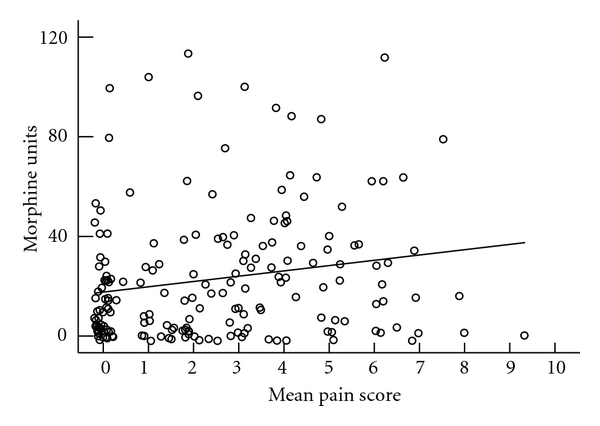
Mean VAS score and morphine PCA utilization (mg).

**Figure 4:**
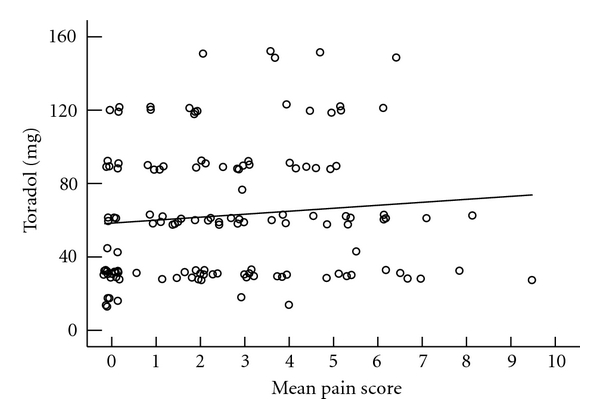
Mean VAS score and frequency ketorolac utilization (30 mg).

**Figure 5:**
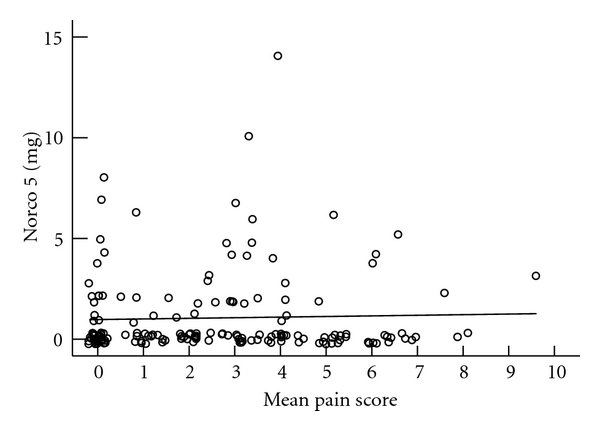
Mean VAS score and frequency Hydrocodone + Acetaminophen 5 mg administered (0 = never administered, 10 = 5 administrations recorded during study participation).

**Figure 6:**
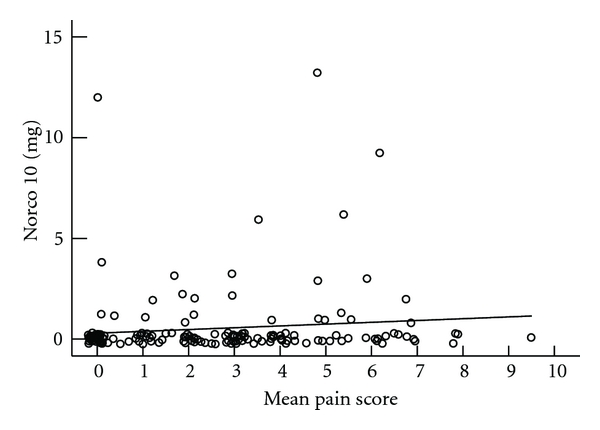
Mean VAS score and frequency Hydrocodone + Acetaminophen 10 mg administered (0 = never administered, 10 = 5 administrations recorded during study participation).

Mean VAS score and pain medication utilization are presented in Figures 3, 4, 5, and 6. We do not see mean VAS scores as predictive of morphine PCA use (*r* ^2^ 0.035), IV nonnarcotic use (*r* ^2^ 0.01), or oral narcotic pain medication use (*r* ^2^  <  .001 for 5 mg, *r* ^2^ 0.012 for 10 mg). The number of participants using other oral narcotics was insufficient for analysis.

## 4. Conclusions

We found that the impact of post operative mean VAS scores was extremely limited, predicting neither pain medication utilization nor frequency of pain assessment in our sample of post operative gynecologic surgery patients. Each patient's interpretation of the VAS score is highly individualistic given difference in pain tolerance and perception. Likewise, decision to accept pain medication are multidimensional and not based on a unidimensional determination of pain severity. Within the context of a well-designed and controlled randomized clinical trial, the utilization of multiple modalities more closely resembles the individualized pain medication strategy as currently advocated. The findings of this study can be generalized to similar populations of gynecologic surgical patients, given distribution of procedure type and demographics. 

Despite the purported utility of VAS scores, we found that the scores did not appear to guide clinical care or optimize pain management. We were surprised that only 49% of our participants had 2 or less VAS scores recorded over the average study time of 18 hours. This may be due to a failure of clinical staff to prioritize VAS scores collection. Although we were surprised by this finding given the emphasis that JCAHO places on the VAS score as the “fifth vital sign,” it was not necessarily representative of poor pain control, as evidenced by the low VAS scores.

VAS utility may be limited by perceptions that it is more difficult and time consuming to assess in comparison to a simple numerical rating scale of 1 to 5 or 1 to 10 [15]. Our findings are particularly interesting in light of the JCAHO study, which showed that patients' actual pain intensity did not relate to perceived overall satisfaction but that patients' overall satisfaction with pain management was predicted by their perceptions of pain assessment frequency, response time, and concern demonstrated by staff [14].

Pain medication utilization did not increase with increased VAS scores. This supports the idea of pain sensation and control as an individualized experience. Neither self- nor RN-administered pain medication utilization was associated with VAS scores. This suggests that patients with higher scores did not use more medication, perhaps secondary to increased pain tolerance than those with lower scores who used the same amount of medication. We did not collect medication administration times, which if linked to VAS time could have provided more insight to when and why patients with consistently low scores were receiving pain medication, and conversely those with higher scores were not receiving higher medication doses.

Our findings provide further support to the literature indicating large variability between individuals in response to noxious surgical stimuli [27, 28]. We suggest that our findings be used to expand assessments of pain in the post operative period to include an understanding of a patient's pain threshold, beliefs about pain relief, decisions to utilize pain medication and response to pain medications. Ideally, customized regimens could be implemented to optimize postoperative pain treatment while minimizing side effects of medication [29].

## Figures and Tables

**Table 1:** Demographic data and surgery type (*N* = 219).

Age (mean)	54 years (SD 14)
Race	
Caucasian	174 (80%)
African american	32 (15%)
Hispanic	3 (1%)
Other (asian, native american)	10 (4%)
Surgery type	
Abdominal	182 (83%)
Vaginal	43 (20%)
Laparoscopic	77 (35%)

*Participants could be categorized as both abdominal and laparoscopic, depending on their procedure.

**Table 2:** VAS score assessment frequency.

Number VAS scores recorded per subject	Frequency
0	14% (31)
1	17% (39)
2	18% (40)
3	19% (41)
4	9% (20)
5	10% (22)
6	3% (6)
>6	10% (20)
